# The Impact of Synovial NF-κB Activation on Apoptosis Pattern Change During Adjuvant-induced Inflammation

**DOI:** 10.18869/nirp.bcn.8.3.173

**Published:** 2017

**Authors:** Sahar Golabi, Jalal Zaringhalam, Homa Manaheji

**Affiliations:** 1. Neurophysiology Research Center, Shahid Beheshti University of Medical Sciences, Tehran, Iran.; 2. Department of Physiology, School of Medicine, Shahid Beheshti University of Medical Sciences, Tehran, Iran.

**Keywords:** Inflammation, Arthritis, NF-κB, Apoptosis, Synovial membrane, Complete Freund’s Adjuvant

## Abstract

**Introduction:**

Apoptosis dysregulation plays a substantial role in the pathophysiology of chronic inflammation and its related symptoms such as edema. Regulation of NF-κB activation is involved in apoptosis pattern change. The current study aimed at verifying the effects of local inflammation on edema and changes in apoptotic markers, and investigating the possible role of NF-κB in apoptosis pattern change during different stages of complete Freund’s adjuvant (CFA)-induced knee arthritis in rats.

**Methods:**

A total of 96 male Wistar rats were divided into different experimental groups. Arthritis was evoked into the right knee articular joint. Changes made in knee edema were assessed by caliper on the days 0, 7, 14, and 21 of the study. Synovial NF-κB and levels of apoptotic markers were evaluated during different stages of the study using Western blot technique.

**Results:**

CFA injection caused intense edema during the whole study period. Synovial NF-κB level increased during the whole study period. The level of apoptotic markers increased during the acute phase of study. But during chronic phase, the apoptosis level decreased. Inh-NF-κB administration increased synovial apoptosis during the whole study period.

**Conclusion:**

It seems that apoptosis pattern change plays an important role in the progression and modulation of CFA-induced inflammation and its related symptoms. Also, it can be concluded that synovial NF-κB had a crucial role in synovial apoptosis change during the study period.

## 1. Introduction

Inflammation is the primary and, essentially, important physiological reaction that normally resolves with the restoration of normal structure and function of tissue. It has symptoms such as edema, warmth, and redness (Akhtari, Zaringhalam, Eidi, Manaheji, & Tekieh, 2012; Foyet et al., 2014). Chronic inflammation is a long-duration inflammation, which can cause tissue damage and loss of function (Akhtari, et al,. 2012; Multag, 2012). Chronic inflammation is also along with increased local and systemic levels of certain inflammatory mediators such as cytokines and chemokines (Zhang, Yin, Xu, Wu, & Chen, 2012; Zaringhalam, Tekieh, Manaheji, & Akhtari, 2013). Rheumatoid Arthritis (RA) is a systemic, chronic inflammatory disorder that affects approximately 1% of the universal population. RA is described by symptoms such as joint swelling, pain, and inflammatory synovitis, which leads to hyperplasia (Kannan, Ortmann, & Kimpel, 2005; Yoshida, Ochiai, Matsuno, Panayi, & Corrigall, 2011; Foyet et al., 2014). Complete Freund’s Adjuvant (CFA)-induced arthritis is an inflammatory model commonly recommended for acute and chronic animal inflammation model (Rezazadeh, Zaringhalam, Manaheji, & Kebryaeezadeh, 2009; Zaringhalam, et al., 2013). This model is valuable to evaluate various behavioral and molecular changes made during acute and chronic inflammation due to its similarity to human RA (Taniguchi, Kanai, Kawamoto, Endo, & Higashino, 2004; Zaringhalam, et al., 2013).

Previous studies demonstrated that apoptosis dysregulation is the main pathophysiological mechanism by which mild prime inflammatory diseases progress to sever chronic inflammatory disorders (Anderson, 1996; Hayashi, 2003). Some studies demonstrated that apoptosis or programmed cell death is a central check-point, which limits the intensity of immune response (Anderson, 1996). Immune system activity and homeostasis are strongly related to apoptosis and chronic inflammation, which can be associated with apoptosis failure (Vandivier, Henson, & Douglas, 2006; Favaloro, Allocati, Graziano, Di Ilio, & De Laurenzi, 2012).

In this regard, studies showed that apoptosis impairment had an important and determinant role in the pathogenesis of lung chronic inflammatory diseases such as Acute Respiratory Distress Syndrome (ARDS), community-acquired pneumonia, Chronic Obstructive Pulmonary Disease (COPD), and Cystic Fibrosis (CF); therefore, in ARDS and community-acquired pneumonia, the number of observed apoptotic cells remain quite low, and in patients with COPD and CF apoptotic cells are increased (Vandivier et al., 2006). Moreover, studies pointed that apoptosis had an important and pivotal role in RA pathogenesis. In this regard, it was revealed that during RA, the synovial fibroblasts were resistant to apoptosis and their resistance correlated with the disease progression. Several anti-apoptotic molecules such as Sentrin -1/small Ubiquitin-like Modifier (SUMO)-1, downstream modulators of Fas signaling and transcriptional regulators such as NF-κB, Stat3, and p53 are nominated to modulate apoptosis in this situation (Baier, Meineckel, Gay, & Pap, 2003; Vandivier et al, 2006; Favaloro et al., 2012; Foyet et al., 2014). Emerging body of proofs suggests that the reconnaissance of better approaches to induce selective apoptosis may lead to new useful therapies for chronic inflammatory diseases (Anderson, 1996).

Furthermore, inflammatory stimuli and inflammation can affect NF-κB activation, as an intracellular signaling molecule (Popivanova et al., 2008), and NF-κB activation is required for most immune and inflammatory responses (Roman-Blas & Jimenez, 2006). Also, studies demonstrated that NF-κB had an important and essential role in chronic inflammatory situations (Roman-Blas & Jimenez, 2006) and inappropriate regulation of its activity was implicated in the pathogenesis of inflammatory diseases such as RA (Roman-Blas & Jimenez, 2006). Moreover, it is described that NF-κB regulates genes involved in apoptosis inhibition and cell proliferation (Roman-Blas & Jimenez, 2006). It was shown that this molecule protects the cell from apoptosis (Haddad & Abdel-Karim, 2011).

Different evidence pointed that steady activation of NF-κB provides a κB involved in the expression variation of numerous inflammatory molecules during synoviocytes. Importance of in vivo NF-κB suppression in the acceleration of inflamed synovium apoptosis was discussed. They showed that in vitro, NF-κB controlled the expression of numerous inflammatory molecules in synoviocytes. Also, they demonstrated that in vivo suppression of NF-κB enhanced apoptosis in the synovium of arthritic rats. It was also found that the severity of arthritis was inhibited significantly in the contralateral untreated joints, which indicated the beneficial systemic effects of local suppression of NF-κB (Miagkov et al., 1998; Makarov, 2000).

Therefore, given the significance of apoptosis pattern change in inflammation pathophysiology, and the important role of NF-κB in inflammation and apoptosis induction, the current study aimed at investigating the synovial apoptosis variation during inflammation and assessing the relationship of NF-κB with this apoptosis variation and the inflammation caused by CFA.

## 2. Methods

### 2.1. Laboratory animals

Adult male Wistar rats, weighing 200 to 220 g were involved in the current study. The animals were kept at 22.0±1°C under a 12-hour light-dark cycle. With the exception of testing time, food and water were available. The current study was conducted align with the investigation guidelines of the international animal models of pain and approved by Shahid Beheshti University of Medical Sciences animal studies local ethic committee (Zimmerman, 1983). The experimental groups were arranged as: CFA; CFA control; CFA+PBS; and CFA+Inh NF-κB. Each group was divided into 4 subgroups to assess the variations during the different days of the study (0, 7, 14, and 21) and each subgroup included 6 male rats.

### 2.2. Local arthritis induction

Arthritis inflammation was induced on the day 0 by a single intra-articular of 100μL CFA (10 mg/mL, CFA, Sigma, St. Louis, MO, USA) injection into the right knee joint of rats. This animal model was selected because it exhibits a rapid primary inflammation response to the adjuvant (Santora, Rasa, Visco, Steinetz, & Bagnell, 2007).

### 2.3. Assessment of CFA-induced arthritis

CIA development was assessed by measuring the knee diameter during different times of the study (on the days 0, 7, 14, and 21). The changes made in the knee diameter on the different days of the study were assessed using a caliper (Multag, 2012).

### 2.4. Synovial tissue extraction

For molecular studies, the knee joint synovial tissue of animals was removed. For this purpose, right knee joint synovia of the animals was opened and synovial membrane was removed carefully from the surrounding enclosures (Hyc, Osiecka-Iwan, Dziunycz, & Moskalewski, 2007). Then, the tissue weight was measured and recorded. At first, the obtained sample was placed in liquid nitrogen for 30 minutes. Then, it was transferred to −80°C freezer for later use.

### 2.5. Measurement of synovial NF-κB and apoptotic markers by Western blotting

Western blot method was employed to measure the synovial level of NF-κB and apoptotic markers (Bax, Bcl2, and Caspase3). The synovial tissue was quickly removed after scarifying the rat and it was homogenized in lysis radioimmunoprecipitation assay (RIPA) buffer. Then, it was centrifuged at 13000 rpm (4°C) for 25 minutes. The supernatant was separated for further analysis and the protein concentration was determined (Bradford, 1976). Equal amounts of proteins were diluted with loading (sample) buffer.

After boiling for 2 minutes, an aliquot of the diluted sample (24 μL) was loaded on electrophoresis gels. Proteins were transferred to polyvinylidene difluoride PVDF membrane (Millipore, Bedford, MA). Non-specific binding sites on the membrane were blocked by incubation in blocking buffer (2% aurora blocking agent) followed by incubation with primary antibody in blocking buffer (rabbit polyclonal IGg for Bax (1/1000), cell signaling; rabbit polyclonal IgG for Bcl2 (1/1000), cell signaling; rabbit polyclonal IgG for Caspase3 (1/1000), cell signaling; and rabbit polyclonal IgG for NF-κB (1/3000), abcam).

Membrane was washed 3 times with TBST (Tris-buffered saline, 0.1% Tween 20), and then, incubated (75 minutes at room temperature) with secondary antibody in blocking buffer (anti-rabbit IgG (1/3000), cell signaling for apoptotic markers; anti-rabbit IgG (1/3000), cell signaling for NF-κB). Membrane was, then, washed 3 times with TBST buffer. The immune reactivity of the proteins on the membrane was visualized using the chemiluminescence detection system (ECL, Amersham). The membrane was, then, incubated in stripping buffer at 37°C and incubated with β-actin primary antibody (rabbit polyclonal IgG for β-actin (1/1000), cell signaling) as a loading control. Band density was measured densitometrically using NIH Image (1.60) and expressed as the ratio of NF-κB, Bax, Bcl2, and Caspase3 bands to β-actin to account for any differences in starting NF-κB, Bax, Bcl2 and Caspase3 proteins. Each experiment was replicated 3 times with new groups of rats.

### 2.6. Experimental procedures

Rats were randomly divided into different experimental groups each of 24 as: CFA, CFA control, CFA+PBS and CFA+Inh-NF-κB. Each group was divided into 4 subgroups to assess the variations during the different days of the study (0, 7, 14, and 21) and each subgroup included 6 male rats. CIA was induced by intra-articular injection of CFA in the right knee joint by light anesthesia on the first day of study. According to the previous studies described for CFA-induced arthritis (Cicala, Innaro, & Fiorucci, 2000; Taniguchi et al., 2004), assessment of knee diameter, NF-κB, and the level of synovial apoptotic markers were conducted on the day 0 (immediately before CFA injection) and on the days 7 (inflammatory phase), 14, and 21 (arthritis phase).

Animals were sacrificed according to the guidelines of animal study ethics at the end of each experiment and synovial membranes were dissected, snap-frozen immediately in liquid nitrogen and kept at −80°C. Synovial NF-κB and apoptotic markers level were assessed by Western blotting on the days 0, 7, 14 and 21 in the experimental groups. This procedure was also applied in the control groups.

### 2.7. Statistical analysis

The results of the statistical analysis were presented as mean±standard error of mean (SEM). To analyze inter-group variations, repeated measurements and one-way analysis of variance (ANOVA) followed by post hoc Tukeys multiple comparison tests in SPSS version 16 were applied. Unpaired student t test was used to determine significant differences in synovial levels of NF-κB and apoptotic markers between the groups. Statistical significance was considered at P≤0.05.

## 3. Results

### 3.1. Knee diameter variation during different stages of the study

**Figure 1.**
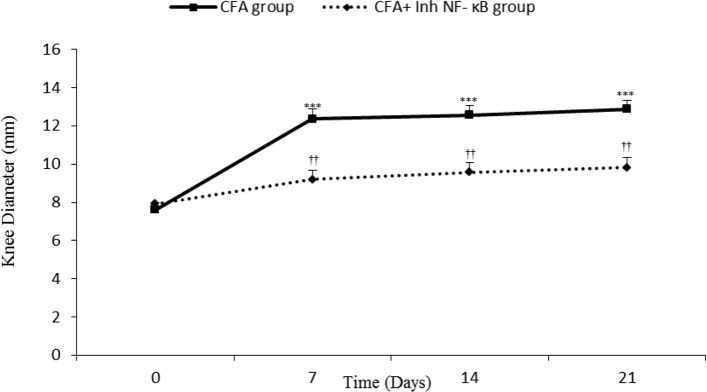
Knee size significantly increased in CFA injected rats. Inh-NF-κB administration significantly changed the inflamed knee size. Data are presented as mean±SEM (n=6/group). ***P≤0.001: Significant difference between the days 7, 14, and 21 compared with the day 0. ††P≤0.01: Comparison of knee size between CFA and CFA+Inh-NF-κB inhibitor treated rats.

Intra-articular CFA injection increased ipsilateral knee diameter, which was continuous until the day 21 of study. Knee diameter meaningfully increased on the days 7, 14, and 21 after inflammation induction when compared with the day 0 (P≤0.001 days). The current study findings in CFA+inh-NF-κB group showed that inh-NF-κB administration caused significant reduction in knee diameter on the days 7, 14, and 21 of the study compared with those of the same days in the CFA control group (P≤0.01) (Figure 1).

There was no significant difference between the CFA injected and CFA+PBS groups in knee diameter variation during different phases of the study (results not shown). Also, there was no significant difference between the CFA injected and CFA control groups in knee diameter variation during different phases of the study (results not shown).

### 3.2. Synovial Bax/Bcl2 variation during different stages of the study

**Figure 2a.**
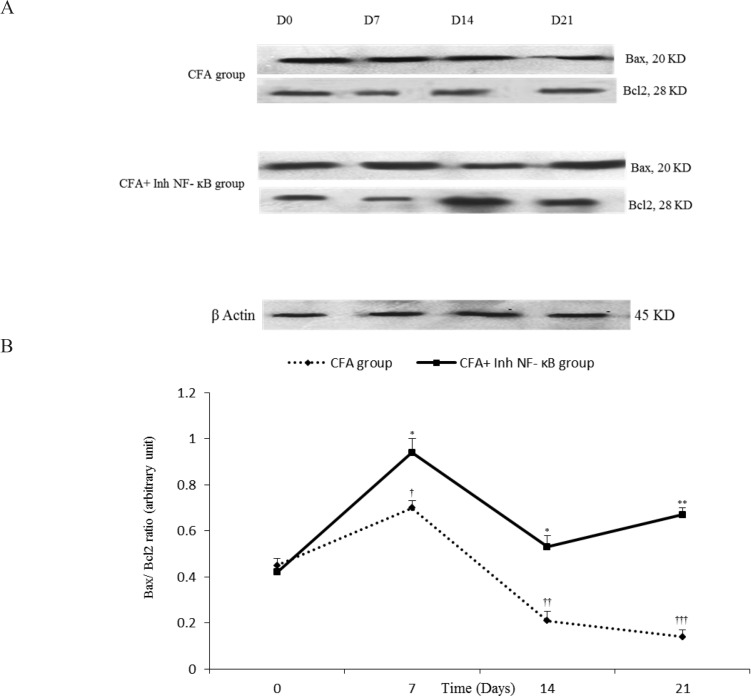
Immunoblots of Bax and Bcl2 extracted from the synovial membranes normalized to β-actin during different stages of the study (the days 0, 7, 14, and 21). Variation of synovial Bax/Bcl2 during different days in CFA and CFA+Inh-NF-κB groups. **Figure 2b.**Synovial Bax/Bcl2 changed in different days of the study compared to the day 0 in CFA group. Inh-NF-κB administration significantly changed synovial Bax/Bcl2. Data are presented as mean±SEM (n=3–4/group). *P≤0.05: Significant difference between the days 7 and 14, and the day 0 in CFA group. **P≤0.01: Significant difference between the days 21 and 0 in CFA group. †P≤0.05; ††P≤0.01. and †††P≤0.001: Comparison of synovial Bax/Bcl2 band intensity between different days in CFA and CFA+ Inh-NF-κB inhibitor treated rats.

Densitometric analysis demonstrated that intra-articular CFA injection time-dependently caused significant changes in synovial Bax/Bcl2 when compared with the control group. The current study results revealed that in synovial membranes obtained from the CFA-injected rats, synovial Bax/Bcl2 significantly increased on the day 7 compared with the day 0 of the study (P≤0.05). On the days 14 and 21 of CFA treatment, synovial Bax/Bcl2 were significantly lower than that of the day 0 (P≤0.05 for the day 14 and P≤0.01 for the day 21). Analysis by densitometry showed that i.p. injection of inh-NF-κB caused a significant increase in synovial Bax/Bcl2 on the days 7, 14, and 21 of the study compared with those of the same days in CFA control group (P≤0.05 for the day 7, P≤0.01 for the day 14, and P≤0.001 for the day 21) (Figure 2a, b).

There was no significant difference between the CFA injected and CFA+PBS groups in synovial Bax/Bcl2 during different phases of the study (results not shown). Based on the current study data, there was no significant difference between the CFA injected and CFA control groups in synovial Bax/Bcl2 during different phases of the study (results not shown).

### 3.3. Synovial Caspase3/β-actin variation during different stages of the study

**Figure 3a.**
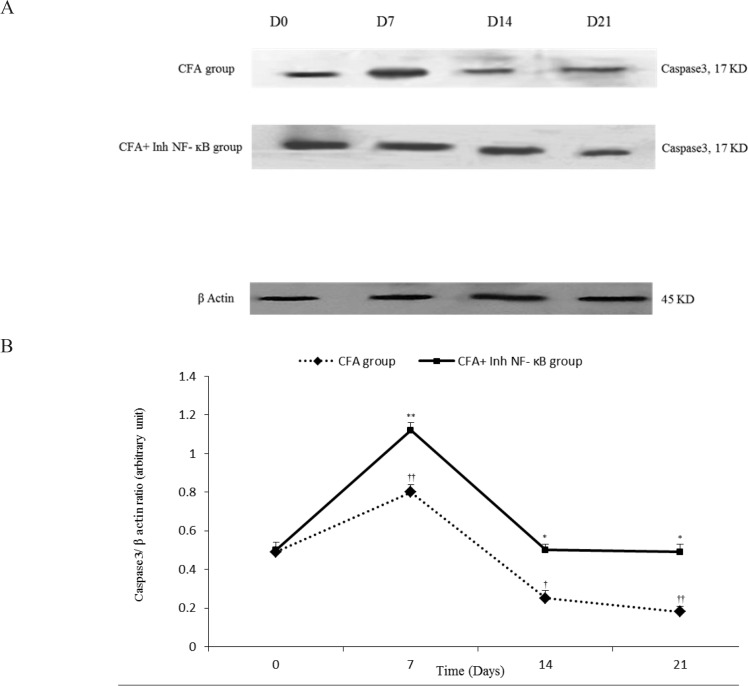
Immunoblots of Caspase3 extracted from the synovial membranes normalized to β-actin during different stages of the study (the days 0, 7, 14, and 21). Variation of synovial Caspase3/β-actin during different days in CFA and CFA+Inh-NF-κB groups. **Figure 3b.**Synovial Caspase3/β-actin changed in different days of the study compared with that of the day 0 in CFA group. Inh-NF-κB administration significantly changed synovial Caspase3/β-actin. Data are presented as mean±SEM (n=3–4/group). *P≤0.05 and **P≤0.01: Significant difference between the days 7, 14, 21, and the day 0 in CFA group. †P≤0.05 and ††P≤0.01: Comparison of synovial Caspase3/β-actin band intensity between different days in CFA and CFA+Inh-NF-κB groups.

The current study results revealed that in the synovial membranes obtained from the CFA-injected rats, caspase3/β-actin significantly raised on the day 7 (P≤0.01) and decreased on the days 14 and 21 compared to the day 0 (P≤0.05 for the day 14, P≤0.01 for the day 21). Western blot analysis showed that inh-NF-κB injection caused a significant increase in synovial Caspase3/β-actin on the days 7, 14, and 21 of the study compared with those of the same days in CFA control group (P≤0.05 for the day 14, P≤0.01 for the days 7 and 21) (Figure 3a, b).

There was no significant difference between the CFA injected and CFA+PBS groups in synovial Caspase3/β-actin during different phases of the study (results not shown). Also, there was no significant difference between the CFA injected and CFA control groups in synovial Caspase3/β-actin during different phases of the study (results not shown).

### 3.4. Synovial NF-κB/β-actin variation during different stages of the study

**Figure 4a.**
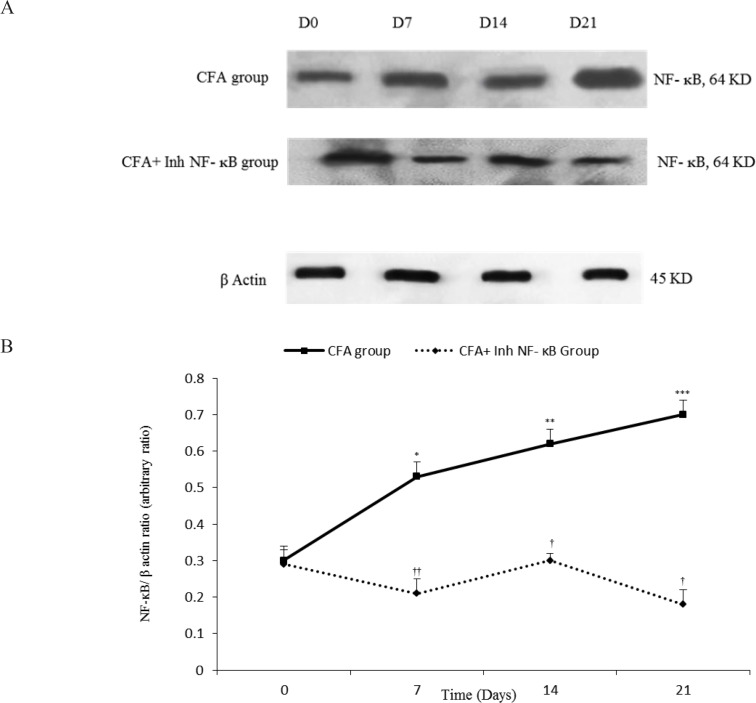
Immunoblots of NF-κB extracted from the synovial membranes normalized to β-actin during different stages of the study (the days 0, 7, 14, and 21). Variation of synovial NF-κB/β-actin during different days in CFA and CFA+Inh-NF-κB groups. **Figure 4b.**Synovial NF-κB/β-actin changed during different days of study compare with that of the day 0 in CFA group. Inh-NF-κB administration significantly changed synovial NF-κB/β-actin. Data are presented as mean±SEM (n=3–4/group). *P≤0.05; **P≤0.01 and ***P≤0.001: significant difference between the days 7, 14, 21, and the day 0 in CFA group. ††P≤0.01 and †††P≤0.001: Comparison of synovial NF-κB/β-actin band intensity between different days in CFA and CFA+Inh-NF-κB groups.

Western blot analysis demonstrated that intra-articular CFA injection caused significant changes in synovial NF-κB/β-actin when compared with those of the control group. The current study findings showed that among the synovial membranes acquired from the CFA injected rats, synovial NF-κB/β-actin significantly increased on the days 7, 14, and 21 when compared with those of the day 0 of the study (P≤0.05 for the day 7, P≤0.01 for the day 14, and P≤0.001 for the day 21). Densitometric analysis showed that inh-NF-κB injection caused a significant reduction in synovial NF-κB/β-actin on the days 7, 14, and 21 of the study compared with those of the same days in CFA control group (P≤0.01 for the days 7 and 14, P≤0.001 for the day 21) (Figure 4a, b).

There was no significant difference between the CFA injected and CFA+PBS groups in synovial NF-κB/β-actin during different phases of the study (results not shown). Also, there was no significant difference between the CFA injected and CFA control groups in synovial NF-κB/β-actin during different phases of the study (results not shown).

## 4. Discussion

The current study results demonstrated that intra-articular CFA injection caused permanent synovial edema. Findings also pointed out that there was a relationship between synovial apoptosis pattern change during the study period and inflammation progression. Also, it was shown that synovial NF-κB had an important role in synovial apoptosis change, which can describe a part of the relationship between CFA-inflammation progress and apoptosis.

CFA injection in rat knee joint caused permanent synovial edema during the whole study period. Studies showed that local CFA injection was one of the most scientific models of inflammatory disorders (like RA) induction and this model was useful to assess various behavioral and molecular changes made during the inflammatory arthritis (Wang & Shuaib, 2002). The authors’ previous studies also showed that plantar CFA injection induced constant inflammation during the 21 days of the study (Akhtari, Zaringhalam, Eidi, Manaheji, Manaheji, & Tekieh, 2015).

On the other hand, the current study findings revealed that CFA-induced inflammation caused an increase in synovial apoptotic markers in the acute phase of the study, but these markers decreased during the chronic phase. The current study results in acute phase of inflammation aligned with those of some previous studies. Among them, it can be pointed that alveolar epithelial cells become apoptotic in acute lung inflammation; therefore, inflammatory mediators in this situation cause apoptosis induction (Matute-Bello, et al., 2001).

Anothers’ study showed that during acute renal inflammation caused by acute ischemic or nephrotoxic insult, apoptosis increases (Lieberthal, 1997). Aligned with the current study results during the chronic phase of inflammation, studies showed that chronic inflammatory bowel diseases can induce cell resistance to apoptosis, and hence, prone the patient to cancer (Shacter & Weitzman, 2002). Previous studies showed that inflammatory disorders can affect apoptosis pattern and also, apoptosis change can affect inflammatory contexts; therefore, apoptosis failure can lead to auto-immune diseases (such as RA), and induction of apoptotic pathways may play important roles in the improvement of inflammatory symptoms (Ramos et al., 2010; Zhang, et al,. 2012).

To the authors’ best knowledge, it was confirmed that inhibition of increased apoptosis in some chronic inflammatory disorders can result in fortification of inflammatory symptoms (Papadakis & Targan, 2000). Moreover, the current study results showed that articular CFA injection caused an increase in synovial NF-κB level during the 21-day study period and Inh-NF-κB decreased knee diameter in the whole study period. It was shown that incorrectly regulation of NF- κB associated with conditions such as autoimmune and inflammatory diseases (Freudenthal, et al., 1998; Merlo, Freudenthal, & Romano, 2002; Meffert, Chang, Wiltgen, Fanselow, & Baltimore, 2003).

Previous studies indicated that NF-κB is one of the principal intra-cellular signaling molecules that mediate inflammatory mediators’ effects (Libermann & Baltimore, 1990; Garcia et al., 2000), and it was also shown that inflammatory situations affect NF-κB activation (Popivanova et al., 2008). Considering the important role of NF- κB in advancing processes that lead to the development of inflammation, it is predictable that inhibition of NF-κB can improve inflammation and its related symptoms such as edema.

Furthermore, the current study findings demonstrated that when CFA induced inflammation, increase in synovial NF-κB level was contrary to the variance of synovial apoptotic markers. Also, the current study showed that inhibition of NF-κB activity caused an elevation in synovial apoptotic markers during the whole study period. These results indicated that NF-κB had an anti-apoptotic effect on synovial tissue during CFA-induced inflammation. Consistent with the current study results, previous studies pointed out the anti-apoptotic effects of NF-κB. It was shown that in human chondrocytes, NF-κB activation mediated anti-apoptotic effects (Roman-Blas & Jimenez, 2006). Also, studies illustrated that defect in NF-κB activity caused increased susceptibility to apoptosis (Haddad & Abdel-Karim, 2011). Accordingly, it seems that the decrease in apoptosis in chronic inflammation is associated with increased activity of NF-κB.

Therefore, according to the results of the current study, a relationship between CFA-induced inflammation and apoptosis variation can be suggested, and it seems that the decrease of apoptotic markers during chronic inflammation can be considered as one of the important reasons of inflammation continuation. On the other hand, the current study results revealed the crucial role of synovial NF-κB in inhibition of apoptosis during chronic inflammation and it seems that treatment by Inh-NF-κB can be suggested as an important method to decrease symptoms of persistent inflammation, which needs more investigations.
